# Motor Signature Differences Between Autism Spectrum Disorder and Developmental Coordination Disorder, and Their Neural Mechanisms

**DOI:** 10.1007/s10803-023-06171-8

**Published:** 2023-12-07

**Authors:** Christiana Butera, Jonathan Delafield-Butt, Szu-Ching Lu, Krzysztof Sobota, Timothy McGowan, Laura Harrison, Emily Kilroy, Aditya Jayashankar, Lisa Aziz-Zadeh

**Affiliations:** 1 USC Mrs. T.H. Chan Division of Occupational Science and Occupational Therapy, University of Southern California, Los Angeles, CA USA; 2 Brain and Creativity Institute, Dornsife College of Letters, Arts and Sciences, University of Southern California, Los Angeles, CA USA; 3 Laboratory for Innovation in Autism, University of Strathclyde, Glasgow, Scotland UK; 4 Faculty of Humanities and Social Sciences, University of Strathclyde, Glasgow, Scotland UK; 5 Faculty of Psychology, University of Warsaw, Warsaw, Poland

**Keywords:** Autism spectrum disorders, Developmental coordination disorder, Machine learning, fMRI, Early detection, Smart tablet

## Abstract

Autism spectrum disorder (ASD) and Developmental Coordination Disorder (DCD) are distinct clinical groups with overlapping motor features. We attempted to (1) differentiate children with ASD from those with DCD, and from those typically developing (TD) (ages 8–17; 18 ASD, 16 DCD, 20 TD) using a 5-min coloring game on a smart tablet and (2) identify neural correlates of these differences. We utilized standardized behavioral motor assessments (e.g. fine motor, gross motor, and balance skills) and video recordings of a smart tablet task to capture any visible motor, behavioral, posture, or engagement differences. We employed machine learning analytics of motor kinematics during a 5-min coloring game on a smart tablet. Imaging data was captured using functional magnetic resonance imaging (fMRI) during action production tasks. While subject-rated motor assessments could not differentiate the two clinical groups, machine learning computational analysis provided good predictive discrimination: between TD and ASD (76% accuracy), TD and DCD (78% accuracy), and ASD and DCD (71% accuracy). Two kinematic markers which strongly drove categorization were significantly correlated with cerebellar activity. Findings demonstrate unique neuromotor patterns between ASD and DCD relate to cerebellar function and present a promising route for computational techniques in early identification. These are promising preliminary results that warrant replication with larger samples.

**Supplementary Information:**

The online version contains supplementary material available at 10.1007/s10803-023-06171-8.

## Introduction

Although not included in the diagnostic criteria, over 80% of individuals with Autism Spectrum Disorder (ASD; autistics) present with noticeable differences in motor functioning as assessed by current instrumentation (Bhat, 2020; Licari et al., 2020; Miller et al., 2021; Zampella et al., 2021). Motor differences have been observed in the neonatal period, suggesting prenatal neurodevelopmental origins (Delafield-Butt & Trevarthen, 2017; Lim et al., 2021; Reynolds et al., 2022; Trevarthen & Delafield-Butt, 2013; Teitelbaum et al., 1998). Similarly, individuals with Developmental Coordination Disorder (DCD) experience impairments in fine motor skill, gross motor skill, dexterity, limb speed, and visual-motor integration (for review see Blank et al., 2019) that present in early development. Unlike ASD, a diagnosis of DCD is not defined by impairments in social functioning, although some secondary social differences may occur as a result of reduced opportunities to engage in sports teams or other social activities (Cermak & May-Benson, 2020). Both children with ASD and DCD display motor disruption in basic and postural motor control, and purposeful movement (Dewey et al., 2007; Eggleston et al., 2017; Lim et al., 2017; Miller et al., 2019; Mostofsky et al., 2006; Paquet et al., 2019; Roley et al., 2014). The early presentation of motor disturbances in both populations suggests an opportunity for the development of early identification tools long before other more perceivable behavioral symptoms arise in ASD.

While assessments of motor skills can be extremely valuable for identifying individuals at high risk for motor delays, more subtle but significant aspects of coordination and timing may be harder to capture (Campbell & Hedeker, 2001). Assessments of motor skills are frequently performed using evaluation measures that are sensitive to human error, time consuming, and measure duration, frequency, and speed of actions involving fine motor, gross motor, and balance tasks (Harris et al., 2015). Other methods, like optical motion capture, can demonstrate disruptions to more subtle kinematic differences, but are expensive, time consuming, and require technical expertise. To mitigate these problems, there has been enthusiasm for utilizing motor game play coupled with machine learning to contribute to diagnosis and understanding of motor dysfunction. Previous literature has shown that it is possible to use machine learning on data from smart tablet motor games or wearable devices to significantly distinguish ASD from TD (e.g. Anzulewicz et al., 2016; Tunçgenç et al., 2021). However, until these digital serious game assessments can provide differential identification between similar childhood disorders (e.g., ASD and DCD), their precise contribution to psychological insight into differences in neurodevelopmental disorders will be limited, as will their potential clinical impact. Therefore, here we aim to identify differences between subtle kinematic motor markers when comparing ASD to DCD using a smart tablet game. Additionally, we use functional Magnetic Resonance Imaging (fMRI) tasks to elucidate underlying neural mechanisms of these kinematic differences in the two clinical groups.

### ASD and DCD Kinematics

In ASD, motor coordination deficits are pervasive (> 80%, Bhat, 2020; Fournier et al., 2010; Licari et al., 2020; Miller et al., 2021), and specific impairments are seen in kinematics for prospective goal-directed movement, but also for gait, posture, and other aspects of motor control (Cavallo et al., 2021; Chua et al., 2022; Eggleston et al., 2017; Miller et al., 2019; Trevarthen & Delafield-Butt, 2013). Synthesis of findings suggest that general sensorimotor integration for the prospective organization of movement is disrupted, and predictive feedforward and feedback mechanisms are consistently impaired (Chua et al., 2022; David et al., 2012; Gowen & Hamilton, 2013; Sinha et al., 2014; Trevarthen & Delafield-Butt, 2013). Deficits in praxis have also been observed in ASD, including poor imitation, gesture to command, and tool use skills (Abrams et al., 2022; Kilroy et al., 2022a, 2022b; Mostofsky et al., 2006; Roley et al., 2014).

Children with DCD commonly display a generalized pattern of deficits in internal modeling, rhythmic coordination, interlimb coordination, gait and postural control, catching, sensoriperceptual function, discontinuous movements, and praxis skills (Paquet et al., 2019; Kilroy et al., 2022a, 2022b). In terms of writing and hand control, children with DCD have difficulties with control in manipulation tasks (Oliveira et al., 2006), hand posture, pen grip force, pen pressure, speed, fluctuations in velocity, and oversized movements (Biotteau et al, 2019).

### ASD vs. DCD Kinematic Motor Differences

Comparing ASD and DCD groups on motor tasks, the results for motor skills such as balance, aiming and catching, and manual dexterity, are mixed, with some studies showing no differences between groups (Kilroy et al, 2022a, 2022b), while others show poorer skills in the ASD group (Dewey et al., 2007; Wisdom et al., 2007), and others show the opposite (Paquet et al., 2019), or mixed results, depending on the motor assessment (Green et al., 2002). However, ASD and DCD may significantly differ in ability to imitate meaningful gestures (ASD performing worse than DCD; Abrams et al., 2022; Dewey et al., 2007; Green et al., 2002; Kilroy et al., 2022a, 2022b; Paquet et al., 2019). Further, there may be differences in the underlying neurobiological basis of motor deficits between the two groups. A previous fMRI study during action imitation, observation, and mentalizing tasks found ASD vs. DCD differences in a number of regions associated with motor planning, sensorimotor functioning, and action understanding (Kilroy et al., 2021).

### Cerebellum in ASD and DCD

Prior studies have shown functional and structural differences in both ASD and DCD groups in the cerebellum (Fatemi et al., 2012; Heijden et al., 2021). The cerebellum has been associated with skills of oculomotor control, motor speech, grip, control of voluntary movement, timing, sensorimotor coordination, and perception of hand movement (Manto et al., 2012); it is also involved in working memory, executive and social functioning, and language processing (Levisohn et al., 2000; Riva & Giorgi, 2000). Cerebellar alterations may be associated with a number of behaviors seen in autism (Sivaswamy et al., 2010), including difficulties with affect processing, executive function, prosody, social skills, eye contact, and repetitive behaviors (Riva & Giorgi, 2000). Studies have demonstrated in autistics, alterations in cerebellar gray matter structure (D’Mello et al., 2015; Stoodley, 2014), disruption of white matter tracts to and from the cerebellum (Catani et al., 2008; Di et al., 2018; Kilroy et al., 2022a, 2022b; Sivaswamy et al., 2010), altered functional connectivity between the cerebellum and the cerebral cortex (Khan et al., 2015; Noonan et al., 2009), and abnormal functional activity in the cerebellum during simple motor tasks (Allen et al., 2004).

Children with DCD also show cerebellar abnormalities, including decreased gray matter volume in cerebellar sensorimotor regions (lobule VIIIa; lobule IX), and an increased gray matter volume in cerebellar regions associated with motor behavior and cognition (lobule VI; crus I and crus II), compared to TD children (Gill et al., 2022b). Compared to controls, children with DCD show reduced activation of the cerebellum during motor tasks of manual dexterity (Fuelscher et al., 2018 for review), predictive motor timing (Debrabant et al., 2013), finger sequencing (Licari et al., 2015), and visuomotor drawing (Pangelinan et al., 2013; Zwicker et al., 2011). Further, in individuals with DCD, a recent study showed gray matter volume increase in the right crus II, left IX, and bilateral VIIIa following an intervention targeted for improving motor performance (Gill et al., 2022a). Taken together, there is ample data suggesting that cerebellar regions may be involved in the interplay between sensorimotor and cognitive processing, and may be relevant to both ASD and DCD symptomology.

### Motor Games

As stated previously, prior studies using standard motor assessments (e.g. Motor Assessment Battery for Children [MABC-2]) find mixed or null results for ASD vs. DCD differences in motor skills. However, such findings may be reflective of measurement issues. One way to mitigate such issues is to use machine learning analysis on more subtle motor information collected during digital motor games using smart tablets or other motion capture hardware. Machine learning has previously been used to analyze children’s movements with an iPad serious game (Anzulewicz et al., 2016), a Kinect dance imitation game (Tunçgenç et al., 2021), and kinematic and eye movement features (Vabalas et al., 2019) to distinguish between ASD and TD children and adults. These studies classified individuals to their respective groups with between 73 and 93% accuracy. Thus, previous findings support the use of machine learning with kinematic data for classifying ASD and TD individuals. However, to make this technology useful, it is essential to be able to distinguish ASD from other neurodevelopmental motor disorders. Here we aim to attempt a similar method to distinguish ASD from another major group of children with developmental motor deficits, those with DCD.

## Methods

### Design

The current study was part of a larger study where youths (aged 8–17; *N*s = 30 ASD, 23 DCD, 33 TD) participated in one day of behavioral testing and a second day of brain imaging (Kilroy et al., 2021). A subset of those youths participated in the current study (aged 8–17; *N*s = 18 ASD, 16 DCD, 20 TD), performing on an iPad serious game following their scan session. Between-group comparisons of the full data set, including behavioral and brain imaging data, can be found in Kilroy et al. (2021), Harrison et al. (2021), Kilroy et al., (2022a, 2022b), Butera et al., (2022, 2023) and Ringold et al. (2022). Here, we include analysis from only the subset that completed smart-tablet games and those with usable brain imaging data (N = 50; 19 TD, 16 ASD, 15 DCD). No autistic people or family members, community providers, policy makers, agency leaders or other community stakeholders were involved in developing the research question, study design, measures, implementation, or interpretation and dissemination of this study.

### Participants

Participants were recruited through flyers and advertisements posted in community centers, on social media, website postings, clinics in the greater Los Angeles healthcare system, and local schools. Exclusion criteria for all groups included (a) IQ < 80 (in the clinical groups, cases where the full-scale IQ was less than 80, participants were included if their verbal IQ score or perceptual reasoning IQ score were greater than 80 as assessed by the Wechsler Abbreviated Scale of Intelligence 2nd edition (WASI-2; Wechsler, 2011); (b) history of loss of consciousness greater than 5 min; (c) left-handedness by self-report or as assessed by a version of the Edinburgh questionnaire (Crovitz & Zener, 1962); (d) not fluent in English or parent without English proficiency; (e) born before 36 weeks of gestation. All participants were screened for MRI compatibility. Inclusion and Exclusion Criteria.

TD controls were additionally excluded if they had any psychological or neurological disorder. Additional exclusionary criteria included: scores below the 25 percentile on the Movement Assessment Battery for Children (MABC-2; Henderson et al., 2007), suspected DCD based on the Developmental Coordination Disorder Questionnaire (DCDQ; Wilson et al., 2007), and a Social Responsiveness Scale, Second Edition (SRS-2; Constantino & Gruber, 2012) score of T > 60, indicating a risk of social impairment. Additionally, a T > 65 on the Conners 3AI-Parent report (Conners, 2008), indicating a risk for attention deficit and hyperactivity disorder (ADHD), was exclusionary for the TD group.

ASD group eligibility included a previous diagnosis through clinical diagnostic interview or diagnostic assessment as well as current clinical symptoms assessed using the Autism Diagnostic Observation Schedule, Second Edition (ADOS-2; Lord et al., 2000), or previous symptoms using the Autism Diagnostic Interview-Revised (ADI-R; Lord et al., 1994). Individuals were excluded if they had a diagnosis of other neurological or psychological disorders with the exception of attention deficit disorder or generalized anxiety disorder. Eight ASD participants were on previously prescribed psychotropic medication at the time of data collection.

Probable DCD group eligibility criteria additionally included: (a) performance at or below the 16th percentile on the MABC-2; (b) no first degree relatives with ASD; (c) no concerns about an ASD diagnosis. The ADOS-2 was administered to participants whose SRS-2 scores were in the “severe risk” category of T = 65–74 (N = 3), but none met ASD criteria so none were excluded. Four DCD participants were on previously prescribed psychotropic medication at the time of data collection.

The study details were relayed in accordance with the protocols approved by the University of Southern California Institutional Review Board, and written child assent and parental consent were obtained. There was no community involvement in the reported study.

### Behavioral Measures

#### Smart-Tablet Game

**Fig. 1:**
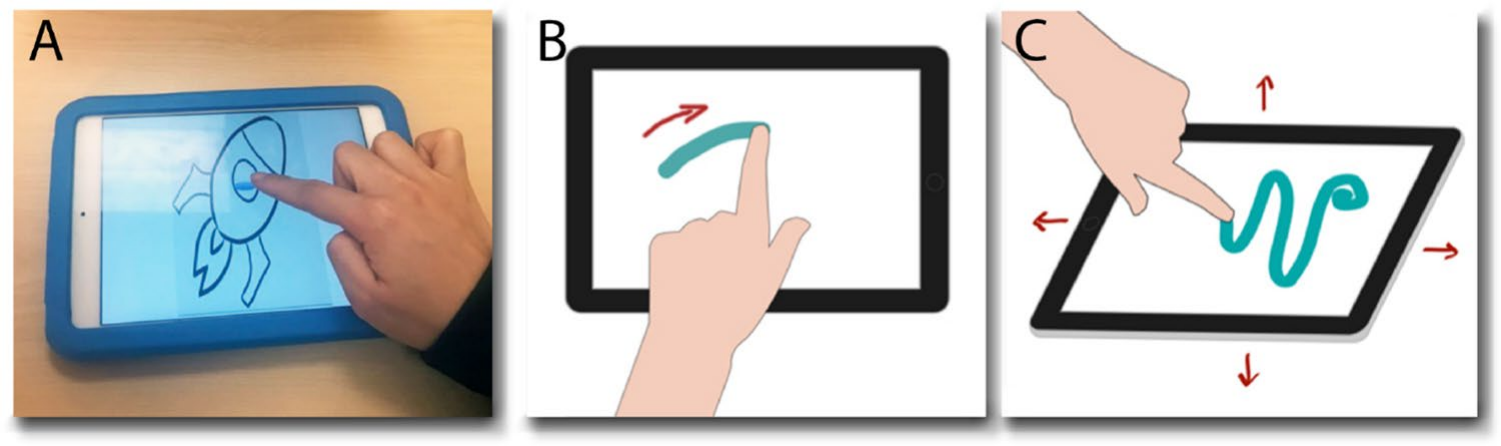
Movement data acquisition. **A** A child engages freely with the smart-tablet game, played on an iPad mini 4. The tablet is protected and made slip-resistant by a bumper and placed firmly on a table. Movement data are acquired from **B** the touch screen and **C** the inertial movement unit (IMU) sensor that detect the touch trajectories and accelerations along with the change of orientation of the iPad resulting from a gesture, respectively. Adapted from Anzulewicz et al. (2016)

A previously-tested naturalistic coloring game (Anzulewicz et al., 2016) was played by participants on an iPad mini (iPad mini 4; iOS 13.3.1). After a two minute trial session to become familiar with available tools and pictures, children completed the coloring game using their dominant hand for 5 min and these spontaneous action patterns were captured by inertial sensor data and touch-screen data from the iPad (Fig. 1).

#### Motor Skills

Motor skills were assessed using the MABC-2 (Henderson et al., 2007), which evaluates manual dexterity, aiming and catching skills, and balance. Subtest and total scores were calculated using the normative samples.

#### Praxis

A version of the The Florida Apraxia Battery (Rothi et al., 2003), modified for children (FAB-M), was used to identify praxis ability and was scoring according to Mostofsky et al., (2006). The assessment includes gesture to command, action imitation, and tool use.

### Behavioral Gameplay Coding Analysis from Video Data

Recordings from a minimum of 55 participants (see Table S1 for full breakdown), made during the 5 min in which kinematic data was captured from tablet gameplay, were coded for posture, engagement, and gameplay behavior. Participants’ activity was coded across 13 characteristics (summarized in Table S1). Two researchers coded the recordings, with 14% of videos being coded by both researchers. Cohen’s Kappa was used to assess inter-rater agreement between researcher’s coding of videos across 12 of the characteristics, whilst the Intraclass Correlation Coefficient (ICC) was calculated for the thirteenth characteristic (‘number of pictures colored’). Coding results for ‘number of pictures colored’ were analyzed using a within-subjects one-way ANOVA, whilst differences between groups within the remaining 12 characteristics were analyzed using Fisher’s exact test.

### Machine Learning Analysis of Drawing Patterns

#### Gameplay and Feature Extraction

Data were collected for analysis from the 5-min assessment phase. Gameplay data were collected by two sets of sensors within the smart-tablet: (1) the touch screen sensor recorded the Cartesian coordinates of each touch, with its displacement across the screen recorded with a variable sampling rate of *ca.* 60 times per second as the finger traveled across the screen; and (2) a triaxial accelerometer and gyroscope inertial movement unit (IMU) sensor that detected the small accelerations and rotations of the smart-tablet device as the child’s fingers impact on the screen, creating small displacements to give subtle, but significant displacive forces during a gesture, with a regular sampling rate of *ca.* 20 times per second. Movement ‘features’ were then calculated from these raw sensor signals to build a computational characterization of each child’s gameplay. These included, for example, for the touch screen sensors, the duration of a gesture, its maximum velocity, deviation from a straight line, its peak acceleration, and the variance of these parameters across a gameplay session. Features from the raw IMU sensors were similarly extracted by calculating, for example, peak acceleration and rotation for each axis, their mean values and SDs.

#### Dataset Preparation and Feature Preprocessing

Two methods were used to extract features for two different forms of machine learning classification. Before employing the machine learning algorithms, data were normalized in the standard feature-wise fashion and only with usage of training data.

First, following previous work (Anzulewicz et al., 2016), we extracted 269 features by simple computations of the raw sensor signals. These were obtained from the touch screen (105 features) and IMU (164 features) data using two approaches: either by calculating the variables for each individual feature or by calculating the variables across the gameplay session. Both were performed using a dedicated, bespoke engine. Touch data for each gaming session were aggregated and split into single, atomic gestures based on the start and end of any particular gesture. For each single gesture, sets of variables were calculated. These features can be split into two major groups: (i) features of movements’ kinematics, (e.g., velocity and acceleration); and (ii) tap-based features, (e.g., the number of taps in a game). Inertial sensor values were computed across the game session irrespective of the touch data. The values for each feature for each game were then reduced to its mean and used as input variables for machine learning training.

The second approach employed to preprocess the touch data again used singular movements, but included more detailed investigation of the profiles of velocity and acceleration of the movements over the movement’s duration (*e.g.* Chua et al., 2022; Lu et al., 2022). Given that each particular movement consisted of a different number of points due to differing gesture durations, each movement was time-normalized (start 0.0 and end 1.0) and split into n time bins to ensure comparability of the movements. After assignment of a bin for each data point, values for each bin were calculated. Simple features were extracted for each bin: mean, median, standard deviation, 25% percentile (Q1), and 75% percentile (Q3). Next, each movement was treated separately (*i.e.* each movement was used as a separate sample during machine learning training, keeping its label to avoid the injection of training set data to test set data). The prediction for this method required averaging the result across all movements made by the user during a gameplay session. Probabilistic output from the model was generated for each movement and saved in the array. The final prediction was calculated as an average of the predictions generated for each movement.

#### Machine Learning Algorithms and Methods of Validation

Given the size of the dataset and imbalance between groups (ratio [1.1–1.25] between groups) several methods were employed to ensure stability of the results and the best possible generalization properties. First, imbalance between groups was mitigated by automatically computing an offset for each classified pair by weights (e.g., the bigger groups were multiplied by a factor less than 0). Next, cross-validation methods were employed with addition of repetitions for k-fold related methods. In the first run, given the small sample size, one-leave-out cross validation was employed. Data were shuffled before each training step. Finally, methods using k-folding were used, both with use of standard and nested approaches (inner loop of cross validation), and to further randomize choice of the fold for validation, the whole process was repeated 10 times. The number of folds was treated as a hyper-parameter. Four folds that produced the best classification accuracy. This method was employed as a best-possible approach to reduce opportunity for over-fitting, but given the small sample size should be considered experimental.

### Machine Learning Differentiation Between ASD, DCD, and TD Groups

Two classes of models were tested. For the combined touch and sensor feature sets, neural networks were primary target models for testing. A typical scheme included usage of shallow networks (two layers deep, with 20–40 units per layer). Adam optimiser (Kingma, & Ba, 2014; Beta 1 = 0.95, Beta 2 = 0.99) with learning rate (0.01–0.001) was used, with addition to invert scaling learning rate (with power = 0.5). Activation function employed: ReLU (Fukushima, 1975; Nair & Hinton, 2010). For the kinematic feature set, a gradient boosting machine learning algorithm was used (Friedman, 2001). A typical scheme employed 500–1000 trees with a learning rate of 0.1, a subsample of 0.8, and with low depth (2) and log2 (feature count) features. Parameters for each model were optimized with a grid search method and the best results selected.

### iPad Feature Analysis

A total of 269 iPad features from each participant were calculated from their smart-tablet gameplay session, including 164 features calculated using the inertial measurement unit (IMU) data and 105 features calculated using the touch trajectories on the screen. The purpose of this analysis was to identify the smart-tablet features statistically different among or between groups for further correlation analyses with the brain imaging data. For those with normal distributions, one-way ANOVA was used to compare the feature values among the TD, ASD, and DCD groups, and an independent-t test was used to compare the feature values between two groups. Except for some testing conditions of the 4 IMU features listed in Table S3, non-parametric tests were performed for comparisons. Kruskal–Wallis test was used to compare the feature values among the TD, ASD, and DCD groups, and Mann–Whitney U test was used to compare the feature values between two groups. These statistical analyses were performed using SPSS.

### Correlation of iPad Features and Brain Imaging ROIs

First, the normality tests of a total of 283 variables were tested, including 269 features and 14 non-feature measures (i.e. age, IQ, MABC-2, and FAB-M scores). The normality of each variable was tested using a one-sample Kolmogorov–Smirnov test across all participants and within each group (TD, ASD, and DCD), and indicated that the data were not normally distributed across participants or within groups (p < 0.05 on most tests). Thus, non-parametric analyses were performed to determine the correlations between each feature and other non-feature measures. Kendall’s tau was chosen because it would be less affected by extreme values. Customized MATLAB scripts were used to perform the correlation analyses.

### Functional Brain Imaging Acquisition and Analysis

Complete information on stimuli, imaging acquisition and analysis can be found in Kilroy et al., 2021, as well as in the *Supplementary Materials*. Below we delineate the action execution and imitation tasks, presented in an 8-min run each, used here. Subjects practiced all tasks in a mock scanner prior to scanning. They were also filmed and monitored in the MRI in order to confirm task completion. In the current study, we used the hand condition (since it is most similar to the iPad task) and a mean of activity across conditions (all conditions; in order to maximize number of trials). A black crosshair in the middle of a white screen was shown for the rest blocks in all runs. Run and block design details may be found in the Supplementary Materials.

*fMRI data acquisition* fMRI data were acquired on a 3 Tesla MAGNETOM Prisma (Siemens, Erlangen, Germany) with a 20-channel head coil. Each functional scan consisted of an echo-planar imaging (EPI; 150 whole brain volumes) acquired with the following parameters: TR = 2 s, TE = 30 ms, flip angle = 90°, 64 × 64 matrix, in-plane resolution 3 × 3 mm, and 41 transverse slices, each 1.5 mm thick, covering the whole brain with a multiband factor of three. Spin Echo EPI field mapping data was also acquired in AP and PA directions with identical geometry to the EPI data for EPI off-resonance distortion correction (TR = 1020 ms, TE1 = 10 ms, TE2 = 12.46 ms, flip angle = 90°, FOV = 224 × 224 × 191 mm3, voxel size = 1.5 × 1.5 × 1.5 mm). A structural T1-weighted MPRAGE was acquired for each subject (TR = 1950 ms, TE = 3.09 ms, flip angle = 10°, 256 × 256 matrix, 176 sagittal slices, 1 mm isotropic resolution). Total scan time was 5 min.

*Within subject analysis* The following preprocessing steps were taken: brain extraction for non-brain removal; spatial smoothing using a Gaussian kernel of FWHM 5 mm; B0 unwarping in the y-direction, standard non-aggressive denoising ICA-AROMA (Pruim et al., 2015) to remove motion-related noise, high pass filter with a cutoff period of 90s, and subject-specific motion correction parameters were entered as nuisance regressors. Functional images were registered to the high-resolution anatomical image using a 7-degrees of freedom linear transformation. Anatomical images were registered to the MNI-152 atlas using a 12-degree of freedom affine transformation, and further refined using FNIRT for nonlinear registration.

Participant head motion was evaluated using the mean relative root-mean square framewise displacement (Jenkinson et al., 2002). Those participants who exhibited extreme in-scanner head motion (> 0.4 mm) were excluded from data analysis. Additional retrospective head motion correction was employed during data analysis. In first-level analysis, individual head motion parameters were included in the GLM. Following which, independent components (from ICA) were filtered using ICA-AROMA. No significant differences in either absolute or relative head motion were found between groups in any task.

#### Execution

Still stimuli were used as cues to execute a pre-trained action. In each 15-s block, three stimuli (5-s each) were presented from one of three categories: emotional facial actions (e.g. sad face as cued by photo of a dead plant), non-emotional facial actions (e.g., tongue to lip as cued by photo of face with whip cream on lip), and bimanual hand actions (e.g., a photo of a xylophone cuing pantomiming playing the xylophone). Participants were instructed to perform the cued action for the entire time that the stimulus was presented (5 s).

#### Imitation

The Imitation task used videos depicting three categories of actions: (a) emotional face actions (e.g., smiling); (b) nonemotional face actions (e.g., tongue to upper lip); (c) bimanual hand actions (e.g., hands playing xylophone; face not shown). Participants were instructed to copy what they saw for the full duration of each video.

#### Within-Subject Analyses

Subject level functional imaging analyses were completed using FSL. For preprocessing and registration procedures, please see Supplementary Materials. Experimental conditions were each modeled with a separate regressor derived from a convolution of the task design and a double gamma function to represent the hemodynamic response and temporal derivative.

#### Group Analysis

All three groups were entered into the multivariate linear regression model. Age, sex, and full scale IQ were centered across groups and entered as covariates. For group analysis, image registration was performed using FSL’s FLIRT (Jenkinson & Smith, 2001; Jenkinson et al., 2002). Each individual’s statistical images were entered into a higher level mixed-effects analysis using FSL’s FLAME algorithm. Three stimulus conditions (emotional face, non-emotional face, and hand actions) were collapsed to determine the main effect of the task compared to a resting baseline. Resulting group level images were thresholded using FSL’s cluster probability algorithm, with Z > 3.1 and a corrected cluster size probability of p = 0.05, FDR.

#### Whole Brain Activation Related to iPad Features

To determine whether smart-tablet features were correlated with BOLD response to the action execution or imitation task, three separate regression analyses were performed with the mean-centered features. These comparisons were also thresholded at Z > 3.1, FDR. Parameter estimates for significant clusters were extracted from each participant and plotted in a graph to rule out the presence of outliers. Any individual who had a mean percent signal change over 3 box lengths (length between first and third quartiles) from the median was removed from feat query analyses, and *R* and *p* values for the whole group and within-group correlations were calculated.

#### ROIs

We focused on anatomical ROIs defined using the Harvard–Oxford dictionary and including the cerebellar crus I and cerebellar crus II, which were binarized and thresholded at 35%. Parameter estimates for ROIs were extracted from all task conditions for the execution and imitation tasks. Independent-samples t-tests were performed to identify group differences in ROI activation. Pearson correlation was performed across groups and within groups between each parameter estimate with the three selected iPad features that demonstrated the group differences in earlier analyses.

## Results

### Behavioral Gameplay Coding

Cohen’s Kappa demonstrated high inter-rater reliability between researchers with κ ranging from 0.579 to 1.00 with a mean of 0.808 across all characteristics excluding ‘number of pictures colored’; for this characteristic there was perfect agreement between researchers (ICC = 1.00). There were no significant differences between groups (F(2, 52) = 0.142, p = 0.868) in the number of pictures colored. Fisher’s exact tests performed for the remaining 12 characteristics showed no significant difference between groups (*p* > 0.08 for all characteristics). Results demonstrate a lack of evidence for visible behavioral, postural or engagement differences during gameplay between participants with ASD, DCD, or TD youths.

### Demographics and Group Differences

Results included 50 participants (18 ASD, 16 DCD, 20 TD, 15 females). Our sample self identified as 48% White, 12% Black, 10% Asian, 16% more than one race, and 13% not reported; with 20% identifying as Hispanic or Latino. Families' self-reported annual household gross income was: 16% less than eighty thousand ($80K), 32% $80K–$140K, 12% $140K–$200K, 10% $200K–$260K, 14% 260K–320K, and 14% more than 400K. All other means and standard deviations are reported below in Table 1. Children with ASD and DCD did not differ on IQ, motor performance, or praxis measures, though both groups differed on motor performance and praxis as compared to TD participants (Table 1).

**Table 1:** Descriptives and group comparisons

	TD	ASD	DCD	TD, ASD, DCD	TD, ASD	TD, DCD	ASD, DCD
	Mean ± SD	Mean ± SD	Mean ± SD	*p*	*p*	*p*	*p*
Age	12.09 ± 2.58	12.36 ± 1.99	12.03 ± 2.25	0.755			
WASI-II VCI	114.85 ± 12.73	109.72 ± 20.27	115.44 ± 17.54	0.339			
WASI-II PRI	111.95 ± 12.42	108.22 ± 20.82	107.56 ± 23.13	0.710			
WASI-II FSIQ-4	115.30 ± 11.22	109.56 ± 19.35	112.38 ± 19.88	0.583			
WASI-II FSIQ-2	115.35 ± 11.07	109.00 ± 17.77	114.44 ± 19.01	0.481			
MABC-2 MD	10.00 ± 2.38	4.56 ± 1.98	5.25 ± 2.60	0.000**	0.000**	0.000**	1.000
MABC-2 AC	11.15 ± 2.96	6.22 ± 3.69	6.44 ± 2.76	0.000**	0.000**	0.001**	1.000
MABC-2 balance	10.40 ± 2.50	6.78 ± 3.46	5.31 ± 2.15	0.000**	0.003**	0.000**	0.328
MABC-2 total	10.40 ± 1.67	4.67 ± 2.45	4.44 ± 1.79	0.000**	0.000**	0.000**	1.000
FAB-M GTC	0.69 ± 0.14	0.57 ± 0.13	0.63 ± 0.15	0.035*	0.029*	0.724	0.575
FAB-M IMI	0.65 ± 0.13	0.46 ± 0.16	0.51 ± 0.17	0.001**	0.001**	0.034*	1.000
FAB-M TU	0.80 ± 0.09	0.59 ± 0.15	0.65 ± 0.14	0.000**	0.000**	0.012*	0.675
FAB-M IMI ML	0.58 ± 0.21	0.40 ± 0.22	0.44 ± 0.20	0.027*	0.032*	0.160	1.000
FAB-M IMI MF	0.70 ± 0.13	0.51 ± 0.17	0.58 ± 0.17	0.002**	0.002**	0.054	1.000

Kruskal–Wallis was first used to test the differences among the three groups. If the distribution was not the same across the three groups (p < 0.05), Dunn’s pairwise tests were carried out for the three pairs of groups. *p < 0.05, **p < 0.01; *** p < 0.001

*WASI-II* Wechsler Abbreviated Scale of Intelligence, Second Edition, *VCI* Verbal Comprehension Index *PRI* Perceptual Reasoning Index, *FSIQ-4* Full Scale IQ, *FSIQ-2 *Two Factor IQ, *MABC-2* Movement Battery for Children, Second Edition, *MD* Manual Dexterity, *AC* aiming and catching, *FAB-M* Florida Apraxia Battery-Modified, *GTC* gesture to command, *IMI* imitation, *TU* tool use, *ML* meaningless gestures, *MF* meaningful gestures, *SD* standard deviation

### Machine Learning Differentiation Between ASD, DCD, and TD Groups

Machine learning analytics of the smart-tablet sensor data were successful in differentiating ASD from DCD motor patterns with 71% accuracy, as well both ASD and DCD as from TD motor patterns with 76% and 78% accuracy, respectively (Table 2). When classified in a 3-way paradigm, which is a more challenging classification task, overall accuracy yield was 57%, or 73% above chance (Table 3).

**Table 2:** Machine learning classification performance between sets of two groups

Pair	Feature set	Accuracy	Sensitivity	Specificity
TD, ASD	Touch, sensors	0.763	0.85	0.67
TD, ASD	Kinematic	0.634	0.65	0.60
TD, DCD	Touch, sensors	0.778	0.75	0.81
TD, DCD	Kinematic	0.666	0.7	0.63
ASD, DCD	Touch, sensors	0.706	0.67	0.75
ASD, DCD	Kinematic	0.694	0.67	0.80

**Table 3:** Machine learning classification confusion matrix for all three groups

		Clinical group		
		TD	ASD	DCD
Machine learning classification	TD	14	2	4
	ASD	5	11	2
	DCD	5	5	6

The confusion matrix shows overall accuracy yield of 57%, which is 73% above chance

### Comparisons Between ASD, DCD, and TD Groups

Statistical results indicated no 3-way significant differences in the feature distributions across all groups. Features with 3-way differences of p < 0.1 between all groups, and significant differences (p < 0.05) between pairwise groups are reported (Table 4). Based on these data, three features (touch features only, less sensitive to artifact of picking up the tablet) were selected for further correlation analyses with brain imaging data: *Gesture Area Variance, Minimum Gesture Acceleration, and Gesture Directness Variance*. *Attitude Variance* was additionally included in machine learning analysis. The three remaining features computed (i) the variance in the area of a gesture of all swipes during a trial, where area was calculated by placing a minimal polygon around the swipe and its area calculated (Gesture Area Variance); (ii) the minimum acceleration of a swipe (Minimum Gesture Acceleration); and (iii) the variance in the smoothness of a gesture during its final data points across a gameplay trial (Gesture Directness Variance).

**Table 4:** Feature data; significant differences between groups

Feature name	Type	TD, ASD, DCD	TD, ASD	TD, DCD	ASD, DCD
		*p*	*p*	*p*	*p*
Attitude variance (x-axis)	IMU	0.058	0.038	–	0.042
Gesture area variance^a^	Touch	0.058	–	–	0.030
Minimum gesture acceleration^a^	Touch	0.073	0.030	–	–
Rate of change of acceleration direction (y-axis)	IMU	0.076	–	–	0.046
Rotation variance (x-axis)	IMU	0.076	0.033	–	–
Attitude variance (y-axis)	IMU	0.094	0.035	–	–
Gesture directness variance^a^	Touch	0.098	–	0.036	–

Features with both borderline significant differences (p < 0.1) across TD, ASD, and DCD groups, and with significant differences (p < 0.05) between groups are summarized in this table

^a^Features were selected for further correlation analyses with the brain imaging data

### Neuroimaging Results

**Fig. 2:**
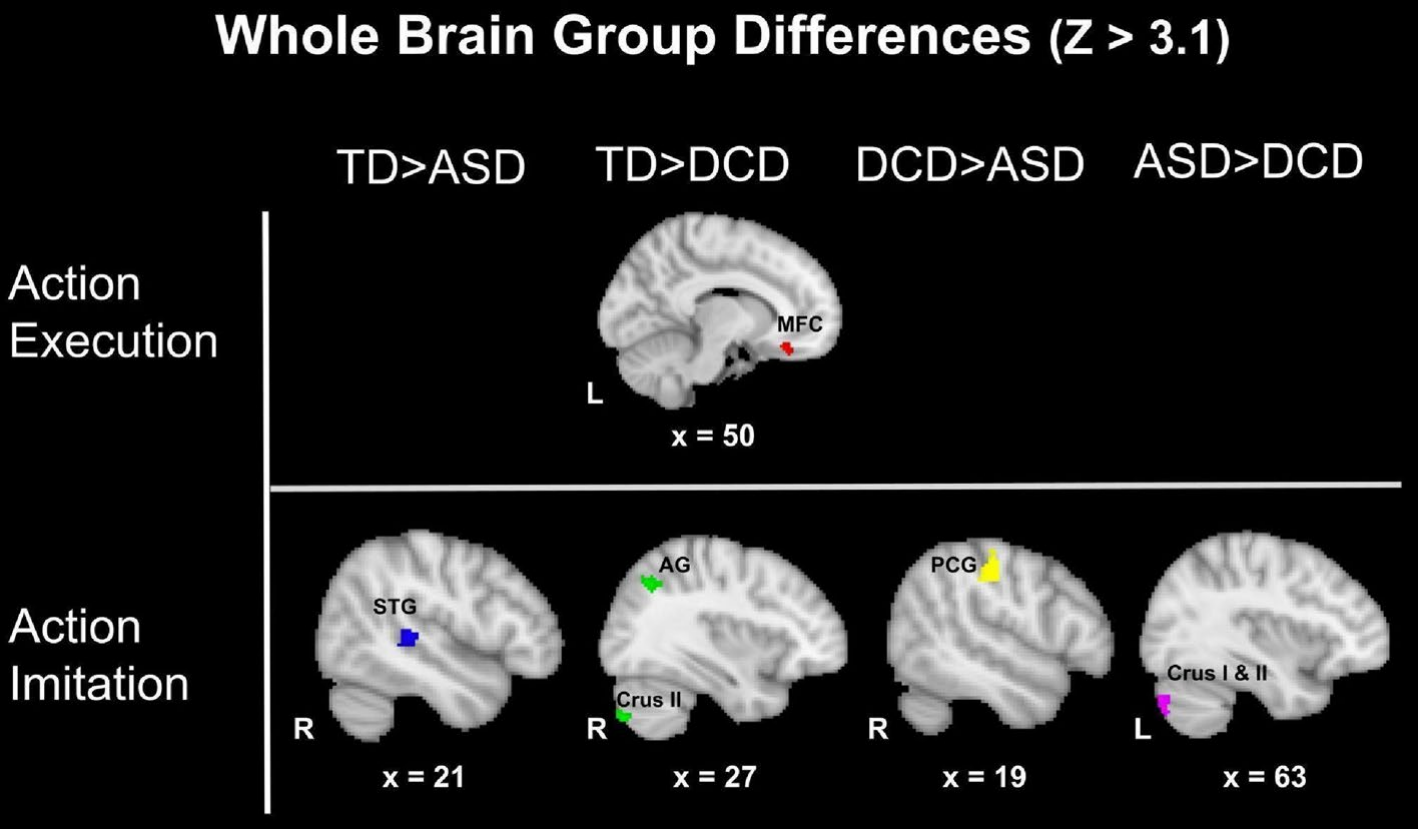
Group differences during all actions. *STG* superior temporal gyrus, *AG* angular gyrus, *PCG* postcentral gyrus, *MFC* medial-frontal cortex, *R* right; *L* left

*Whole Brain Group Differences* In the *execution task*, the only significant group difference identified was TD > DCD in the medial-frontal cortex during the all action condition (Fig. 2). During the *imitation task*, DCD demonstrated a decrease in cerebellar activation when compared to both TD (right cerebellar crus II) and ASD (left cerebellar crus I & II) groups during all conditions (Z > 3.1; Fig. 2). During imitation of hand actions, the DCD group had reduced activation compared to the TD group in right lateral occipital cortex, and the angular gyrus during all conditions (Z > 3.1; Fig. 2), and in the right lateral occipital cortex, and the left frontal pole (Z > 3.1). During imitation of all actions, the ASD group had lower activation of the right postcentral gyrus compared to the DCD group, and of the right precuneus and right superior temporal gyrus compared to the TD group **(**Z > 3.1; Fig. 2).

#### ROI Group Differences

**Fig. 3:**
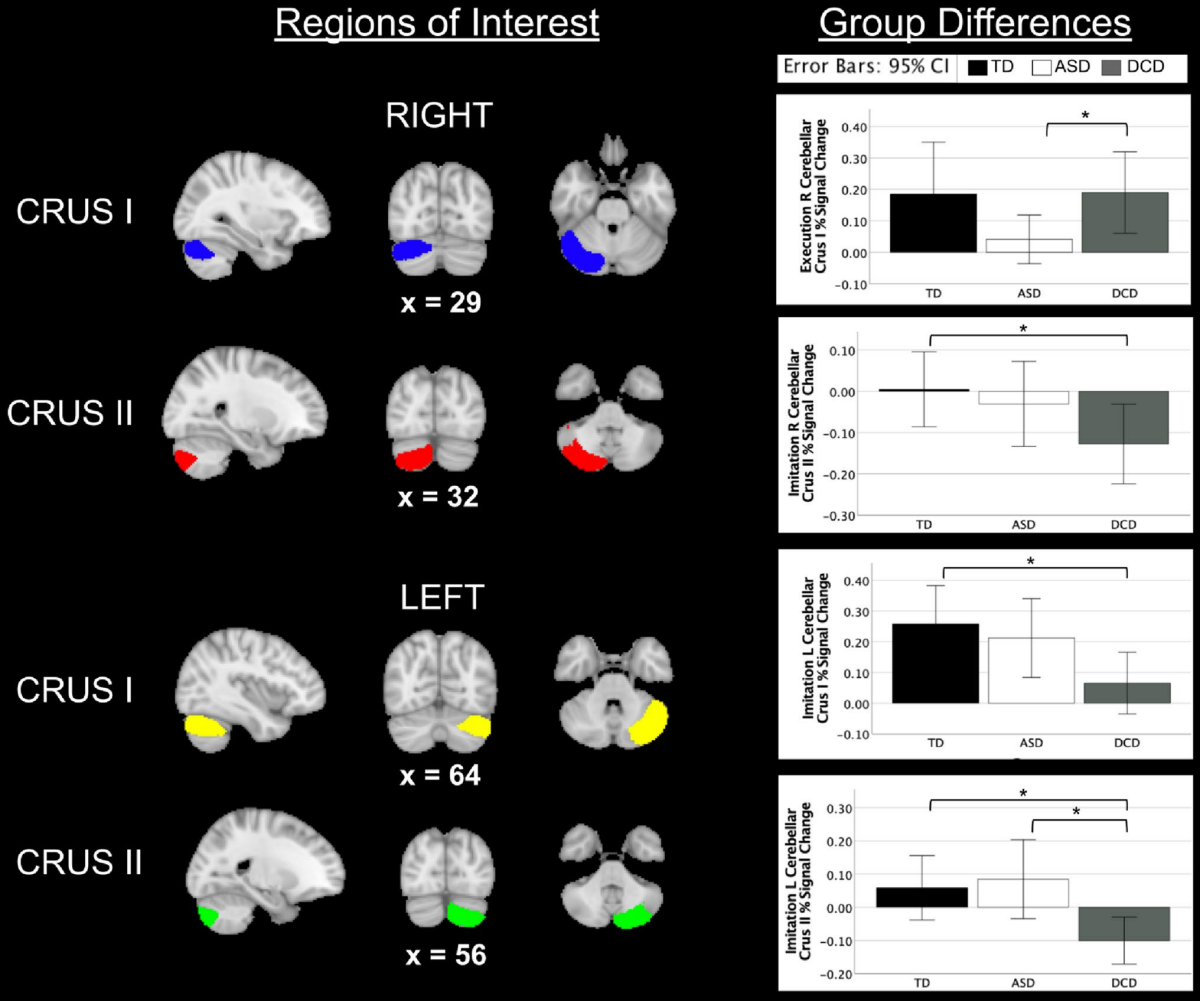
Regions of interest and group differences. Between group differences in cerebellar regions of interest during execution and imitation of all actions. *TD* typically developing, *ASD* autism spectrum disorder, *DCD* developmental coordination disorder, *R* right, *L* left

Visuals of ROIs and group differences can be found in Fig. 3. During execution of all actions, the ASD group had reduced activation in the right cerebellar crus I compared to the DCD group (p = 0.033). During action imitation, the DCD group had reduced activation compared to the TD group in left cerebellar crus I (HAND: *p* = 0.015; ALL: *p* = 0.020), the right cerebellar crus II (HAND: *p* = 0.022; ALL: *p* = 0.022), and left cerebellar crus II (ALL: *p* = 0.012). The DCD group also had reduced activation compared to the ASD group in left cerebellar crus II during imitation of all actions (*p* = 0.009).

#### Whole Brain Correlations

**Fig. 4:**
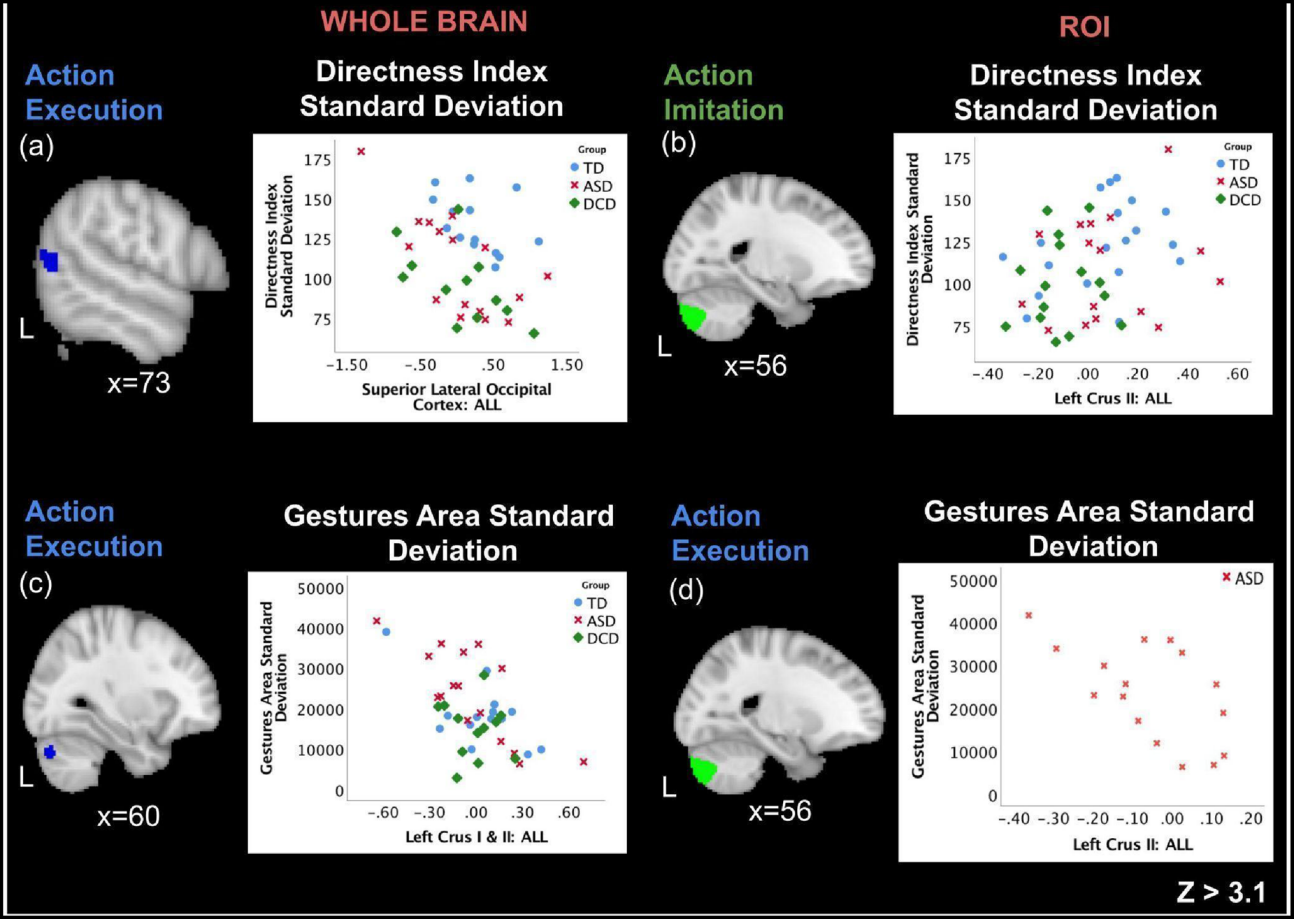
Correlations with iPad features. Correlations during action execution and action imitation across all participants during all action conditions

We correlated the previously mentioned features that distinguished groups (Gesture *Area Variance, Minimum Gesture Acceleration, Gesture Directness Variance*) with levels of change in neural activation during action execution in the scanner. Across all participants during execution of all actions, *Directness Variance* was negatively correlated with activation in the left superior lateral occipital cortex, and *Gesture Area Variance* was negatively correlated with activation in the left cerebellar crus I and II (Fig. 4). Significant correlations were also observed between *Gesture Directness Variance* and left superior lateral occipital cortex in the ASD (r = − 0.693, p = 0.003) and DCD groups (r = − 0.589, p = 0.044). *Gesture Area Variance* was negatively correlated with activation in the left cerebellar crus I and II, during execution of all conditions (Fig. 4). This relationship was also significant in the ASD (r = − 0.765, p = 0.001) group. There were no whole brain correlations in the imitation task with any of the smart-tablet features.

### ROI Correlations

Across all participants, a significant negative correlation was observed between gesture area variance feature and the right cerebellar crus II during execution of all actions (r = − 0.360, p = 0.019). This relationship was also observed in the ASD group where it trended toward a significant negative correlation with the right cerebellar crus II for all conditions (r = − 0.497, p = 0.050). Activity in the left cerebellar crus I was significantly negatively correlated with *Gesture Area Variance* (r = − 0.360, p = 0.019) during execution of hand actions across groups, though this relationship was not significant in any individual group. During imitation, for all actions across groups, the left cerebellar crus II was significantly positively correlated with *Gesture Directness Variance* (r = 0.284, p = 0.046), though this relationship was not significant in any individual group.

## Discussion

Here we demonstrate that using machine learning from a simple motor coloring game on a smart-tablet, we can significantly differentiate the gameplay of children with ASD, DCD, and TD. This is especially significant since in the current study, standard behavioral motor measures could not distinguish between ASD and DCD groups, nor could video coding analysis. We further show that measures that reflect control of movements and their degree of displacement are the driving motor features that differentiate clinical groups. Finally, cerebellar regions previously associated with reduced activation in both ASD and DCD groups, show significant relationships with kinematic features from the smart-tablet data. We further discuss each of these results below.

### Classifying ASD/DCD/TD by Game-Play

Coupled with machine learning, kinematics recorded from the smart tablet game were able to categorize ASD from TD at 76%, ASD from DCD at 71%, and DCD from TD at 78% accuracy. To our knowledge, this is the first time serious game digital technology has been used to distinguish two similar motor developmental disorders—ASD from DCD. Given that visual behavioral analysis of video data nor standard motor assessments, such as the MABC-2 did not distinguish the two groups apart, this finding is especially promising, and suggests that this method may usefully contribute to clinical diagnosis, as well as better informing the particular underlying motor disturbances in each group, although more research with larger sample sizes are needed. Refinements of this technique can be explored in future studies to increase between-group categorization accuracy, for example by additionally including social motor games.

### Motor Markers that Distinguish Groups

The kinematic markers that most contribute to differentiating between groups include the control of deceleration and variability in the distance, or area covered, of the motor gestures. On average, autistics were more variable in the size of the gesture area used on the smart-tablet than individuals with DCD for each motor gesture. This suggests that for an individual with ASD, there is more variability in gesture size, with some gestures made as very small and some as very big in the course of the coloring game, compared to individuals in the DCD group. Such large variation may be related to two contrasting types of gesture behavior within an ASD individual, large gestures driven by a reluctance to shift from the ongoing gesture once engaged with it, and very short gestures produced by rapid tapping. Either way, the underlying nature of ‘restricted and repetitive’ behaviors manifests in each type of motor behavior. Future work will need to investigate this to better understand individual action patterns and their distribution in autistics.

Finally, we investigated neural regions (cerebellar crus I/II) previously associated with differences in ASD and DCD groups (Allen et al., 2004; Gill et al., 2022b; Fuelscher et al., 2018; Debrabant et al., 2013; Licari et al., 2015; Pangelinan et al., 2013; Zwicker et al., 2011), and differences in imitation and praxis (Dapretto et al., 2006). In ASD groups, crus I has previously been shown to be involved in control of hand movements, performance of precision grips (Neely et al., 2013; Vaillancourt et al., 2006), and force variability (McKinney et al., 2022) and related to repetitive behaviors in females (McKinney et al., 2022). Crus I and II together are involved in sensorimotor tasks, as well as working memory, attention, and social cognition (Guell & Schmahmann, 2020; McKinney et al., 2022; Van Overwalle, 2020). Thus they may be particularly involved in the interplay between sensorimotor function and cognition. Notably, while difficulties with working memory, attention, and social cognition are common symptoms of ASD, individuals with DCD may fall between ASD and TD groups on all these behaviors (Kilroy et al., 2022a, 2022b; Ringold et al., 2022). Our data indicate that during motor imitation, the crus II is significantly hypoactive in DCD (Left: TD/ASD > DCD [ROI]; ASD > DCD [whole brain]; Right: TD > DCD [ROI and whole brain]). For the right crus I, during the execution task, we find the ASD group is hypoactive compared to the DCD group (DCD > ASD [ROI]). For the left crus I, during imitation, both clinical groups are hypoactive compared to TD, though the DCD group may show significantly more hypoactivity (TD > ASD/DCD [ROI] and ASD > DCD [whole group]). Taken together, these data indicate that during motor tasks, the right and left crus II are particularly hypoactive in DCD, while activity patterns in crus I may be more nuanced between groups. It is possible that differences previously observed in DCD in imitation performance may be more related to cerebellar influences, rather than imitation differences previously observed in ASD, which may be more dependent on frontal cortical regions (Kilroy et al., 2021).

Interestingly, we find that during our fMRI motor tasks, activity in these cerebellar regions correlates with the iPad kinematic features that are the best at differentiating between specific pairwise groups. During motor tasks, we find activity in the left crus II correlates with *Gesture Directness Variance* across participants and with *Gesture Area Variance* in the ASD group. The latter pattern is also found for the left crus I and right crus II; during execution, activity in these areas correlates with *Gesture Area Variance* across groups and within the ASD group. These cerebellar regions may show differential activation patterns in ASD and DCD, and their activity may also be related to motor control of deceleration and measures of gesture size, which both clinical groups perform differentially, in alignment with prior studies (McKinney et al., 2022). Thus differential activity in these cerebellar regions may lead to behavioral motor differences between groups, allowing the use of kinematic patterns to distinguish between ASD, DCD, and TD groups. Interestingly, only features that were associated with classifying ASD vs DCD, and TD vs DCD differences were correlated with brain activity during our tasks, no features that were best associated with classifying TD vs ASD differences were correlated with brain activity, reiterating the idea that crus I and II may be more involved gesture execution and imitation deficits seen in the DCD group. Previous literature has shown hyperconnectivity (during resting state and motor tasks) between crus I and II and premotor and motor cortices (Jung et al., 2014; Verly et al., 2014), therefore domain specificity of cerebro-cerebellar connections might be abnormal in ASD, rather than cerebellar activation alone.

### Limitations

We note that future studies are needed with larger sample sizes and more diverse groups (e.g., more females; larger age range; left handers; wider range of IQ). Despite machine learning methods employed to reduce overfitting and deliver best-possible results, the small sample size necessarily implies the findings reported here should be tested for replication within larger cohorts. Further, motor games with more social aspects (e.g., imitation, social interactions) may offer even better categorization accuracy between groups. Finally, to better understand the neural mechanisms, future studies may attempt to execute smart-tablet tasks during fMRI, and should probe relationships with other areas of interest that appeared in the whole-brain comparisons (medial-frontal cortex angular gyrus, lateral occipital cortex, left frontal pole, right postcentral gyrus, right precuneus and right superior temporal gyrus).

### Conclusions

Here we show that kinematics from a simple motor smart-tablet game can be utilized to categorize ASD, DCD, and TD groups. We further show that two driving kinematic markers for this categorization are control of deceleration and variability in gesture size. These two kinematic markers are associated with neural activity in cerebellar regions during motor tasks across groups. These data may be important for the development of motor markers for screening and diagnosis of ASD and DCD and for development of individualized interventions.

## Electronic Supplementary Material

Below is the link to the electronic supplementary material.

Supplementary Material 1 (PDF 210 KB)
