# Supplementary material for: Motor Signature Differences Between Autism Spectrum Disorder and Developmental Coordination Disorder, and Their Neural Mechanisms
Source: J Autism Dev Disord. 2023 Dec 7;55(1):353–68. doi: 10.1007/s10803-023-06171-8 (PMC11802596; doi:10.1007/s10803-023-06171-8)
Supplement: Supplementary file 1 — Supplementary Material 1 (PDF 210 KB) [file 10803_2023_6171_MOESM1_ESM.pdf]

Supplementary Materials

fMRI Methods

Stimuli were presented using MATLAB with the Psychophysics Toolbox (Piven et al., 1997). Tasks were eight minutes long including instructions. Stimuli for each condition were presented for 3.75 seconds in a block design consisting of three stimuli per block with a 1.25-second black screen as a transition between each stimuli followed by a 15-second rest. During the rest blocks, participants were shown a black crosshair in the middle of a white screen. Five blocks of each action condition were alternated with rest in a pseudo-random sequence. Seven different adult actors were used to create the stimuli. No video was repeated and no block contained more than two same sex actors. Each participant's tasks began with an additional hand action condition block to habituate participants to the task which was later removed from the analysis.

Execution. The emotional facial actions were cued in the following way: a photograph of a dead plant cued making a sad expression, a photograph of moldy bread cued making a disgust expression, and a poison bottle cued making a fear expression. The non-emotional facial actions were cued in the following way: a spot of whip cream on one of three points around the mouth of a face with a neutral expression cued bringing the tongue to lip in that spot. Bimanual hand actions were cued by: a photograph of a xylophone cuing pantomime playing xylophone, a photograph of grapes cuing pantomiming pulling grapes from stems, and a photograph of a game controller cuing pantomiming pushing buttons on the game controller as if playing a video game.

Imitation. Excluding an initial junk block, five blocks of each stimulus condition were alternated with rest in a pseudo-random sequence creating a total of 15 different videos for each category per run. Seven different Caucasian adult actors were used to create the stimuli. No stimulus was repeated in the same run and no block contained more than two same sex actors.

No more than two videos per block contained the same valenced emotion (i.e., two high valenced emotions and one low valenced emotion).

Table S1. Video Coding

Postural or Behavior Characteristic	Coding Options Within Characteristic	Number of Participants Included for Analysis
Hand used for gameplay	Left	57 (ASD (n = 19, 3 female), DCD (n =18, 7 female) and TD (n = 20, 6 female))
	Right	
	Combination of both hands	
Primary fingers/digits used for gameplay	Thumb	57 (ASD (n = 19, 3 female), DCD (n =18, 7 female) and TD (n = 20, 6 female)
	Index finger	
	Middle finger	
	Ring finger	
	Little finger/Pinkie	
	Combination of fingers and thumb	
Crossing the screen midline whilst tracing and coloring	Midline not crossed	57 (ASD (n = 19, 3 female), DCD (n =18, 7 female) and TD (n = 20, 6 female))
	Midline crossed	
Position of interaction hand during gameplay	Resting on the iPad screen	57 (ASD (n = 19, 3 female), DCD (n =18, 7 female) and TD (n = 20, 6 female))
	Resting on the bumper surrounding iPad	
	Above, not touching iPad	
	Side, not touching iPad	
	Combination of touching iPad screen and above	
	Combination of touching bumper surrounding iPad and above	
Nature of finger movement whilst tracing item	Tracing along guide lines	56 (ASD (n = 19, 3 female), DCD (n =17, 7 female) TD (n = 20, 6 female))
	Scribbling, crossing over guide lines	
	Combination of tracing and scribbling	
Nature of finger movement whilst coloring item	Stroking screen	57 (ASD (n = 19, 3 female), DCD (n =18, 7 female) and TD (n = 20, 6 female))
	Controlled scribbling on the screen	
	Uncontrolled scribbling	
	Combination of both stroking and controlled scribbling	
Area of screen used whilst coloring	Only within the item traced	56 (ASD (n = 19, 3 female), DCD (n =18, 7 female) and TD (n = 19, 5 female))
	Outside of the traced lines, but close to item	
	Whole screen	
	Only item traced and adding to the picture	
Origin of movement generation during gameplay	Movement generated from wrist only	55 (ASD (n = 17, 3 female), DCD (n =18, 7 female) and TD (n = 20, 6 female))
	Movement generated from elbow only	

	Movement generated from shoulder only	
	Movement generated from wrist and elbow	
	Movement generated from wrist and shoulder	
	Movement generated from elbow and shoulder	
	Movement generated from a combination of all arm areas	
Body posture during gameplay	Upright	57 (ASD (n = 19, 3 female), DCD (n =18, 7 female) and TD (n = 20, 6 female))
	Asymmetric, whole body leaning to one side	
	Asymmetric, only head leaning to one side	
	Slouching forward	
	Slouching sideways leaning onto the table	
	Combination of asymmetric leaning and slouching (whole body)	
	Combination of asymmetric leaning and slouching (head only)	
Position of non-interaction hand with iPad during gameplay	Touching screen only to play game	57 (ASD (n = 19, 3 female), DCD (n =18, 7 female) and TD (n = 20, 6 female))
	Holding the bumper, left side	
	Holding the bumper, right side	
	Holding the bumper, top	
	Holding the bumper, bottom	
	Holding the bumper in several areas	
Movement of iPad during gameplay	No movement	57 (ASD (n = 19, 3 female), DCD (n =18, 7 female) and TD (n = 20, 6 female))
	iPad moved once	
	iPad moved twice	
	iPad moved three times	
	iPad moved four times	
	iPad moved 5 times or more	
	iPad moved 10 times or more	
Types of interaction between participant and researcher	No interaction	57 (ASD (n = 19, 3 female), DCD (n =18, 7 female) and TD (n = 20, 6 female))
	Intermittent, relevant interaction	
	Constant, relevant interaction	
	Intermittent, irrelevant interaction	
	Constant, irrelevant interaction	
	Constant, combination of relevant and irrelevant interaction	
Number of pictures colored	1	55 (ASD (n = 19, 3 female), DCD (n =17, 7 female) and TD (n = 19, 5 female))
	2	
	3	
	4	
	5	
	6	
	7	

Table S2. Normality analysis

Feature name	TD	ASD	DCD
AttitudeCorrelation_0_2	***p=0.125***	***p=0.129***	***p=0.216***
AttitudeCorrelation_2_0	***p=0.125***	***p=0.129***	***p=0.216***
AccelerationCorrelation_0_1	***p=0.059***	p<0.05	***p=0.147***
AccelerationCorrelation_1_0	***p=0.059***	p<0.05	***p=0.147***
JerkCorrelation_0_1	p<0.05	p<0.05	***p=0.100***
JerkCorrelation_1_0	p<0.05	p<0.05	***p=0.100***
AttitudeCorrelation_1_2	p<0.05	***p=0.161***	p<0.05
AttitudeCorrelation_2_1	p<0.05	***p=0.161***	p<0.05

Note. One-sample Kolmogorov-Smirnov test was performed for each variable. Of the total 283 variables, 8 IMU feature variables returned p-values >0.05 (i.e. normally distributed). The normality of each feature was tested within each group (TD, ASD, and DCD) to decide if a parametric or non-parametric test should be used to compare the feature values among/between groups. As described in the previous analysis, one-sample Kolmogorov-Smirnov test was performed using customised MATLAB scripts. For most of the features, a p-value <0.05 was returned, which means the test rejected the null hypothesis that the data come from a standard normal distribution. Those with a p-value >0.05 (i.e. normally distributed) are listed in Table 1.

Table S3. Statistical analysis overview

Feature name	TD, ASD, DCD	TD, ASD	TD, DCD	ASD, DCD
AttitudeCorrelation_0_2	***One-way ANOVA***	***Independent-t***	***Independent-t***	***Independent-t***
AttitudeCorrelation_2_0	***One-way ANOVA***	***Independent-t***	***Independent-t***	***Independent-t***

AccelerationCorrelation_0_1	Kruskal-Wallis	Mann-Whitney U	***Independent-t***	Mann-Whitney U
AccelerationCorrelation_1_0	Kruskal-Wallis	Mann-Whitney U	***Independent-t***	Mann-Whitney U

Note. Parametric analyses (one-way ANOVA and independent-t test) were performed on some of the testing conditions of the 4 IMU features listed in this table. Kruskal-Wallis and Mann-Whitney U tests were used for other comparisons of non-normally distributed variables.

Figure S3.

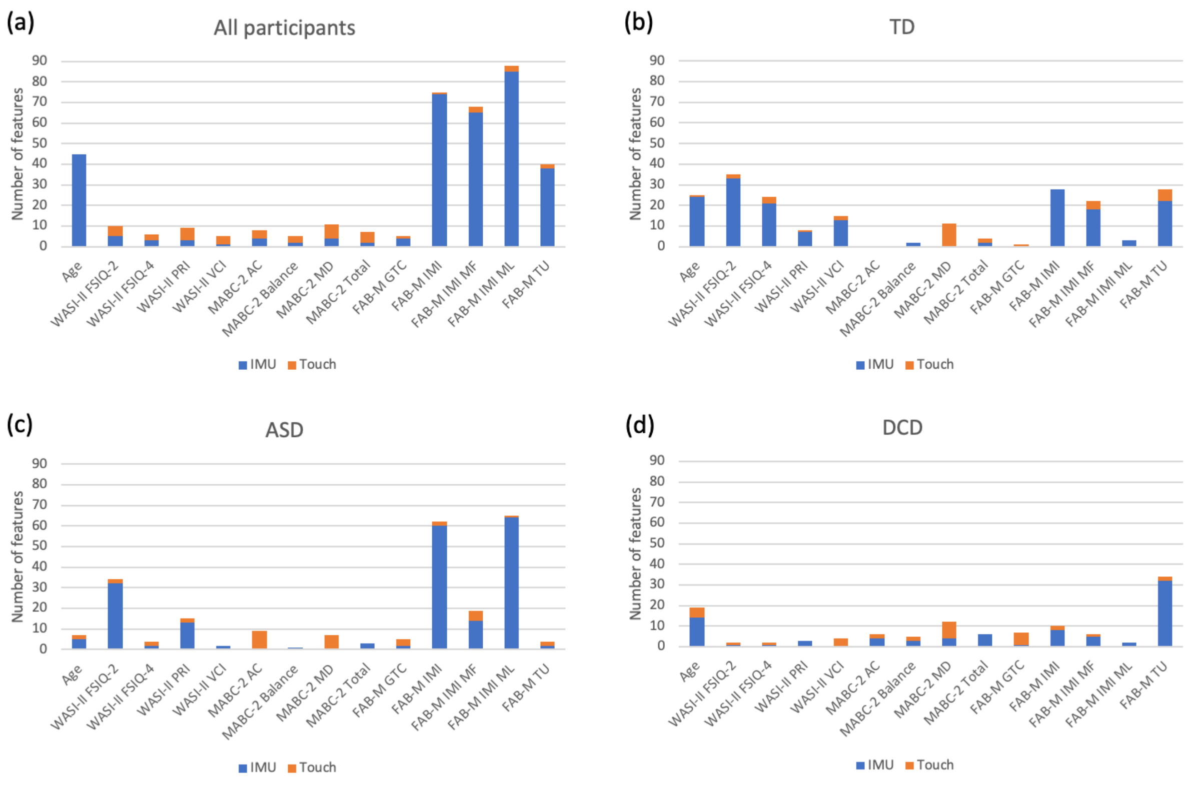

Figure S3. This figure demonstrates the numbers of significant correlations between feature and non-feature variables across all participants (a) and within each group (b-d). Abbreviations: WASI-II = Wechsler Abbreviated Scale of Intelligence, Second Edition; FSIQ-2 = Two Factor IQ; FSIQ-4 = Full Scale IQ; PRI = Perceptual Reasoning Index; VCI = Verbal Comprehension Index; MABC-2 = Movement Battery for Children, Second Edition; AC = Aiming and Catching; MD = Manual Dexterity; FAB-M = Florida Apraxia Battery-Modified; GTC = gesture to command; IMI = imitation; MF = meaningful gestures; ML = meaningless gestures; TU = tool use.

iPad feature analysis I. Correlations with other non-feature measures

The purpose of this analysis is to find the correlations between each feature and other non-feature measures, including participant age, four IQ scores (verbal, performance, full scale-4, full scale-2), four MABC-2 (manual dexterity, aiming and catching, balance, total standard score), and five Praxis scores (gesture to command, tool use, imitation, imitation of meaningful gestures, imitation of meaningless gestures).

A total of 382 significant correlations ($p<0.05$) were observed across all participants (Fig. S3a); 206 significant correlations were found in the TD group (Fig. S3b); 237 significant correlations were in the ASD group (Fig. S3c); and 118 significant correlations were in the DCD group (Fig. S3d). Correction methods to control for the large number of variables, such as the Bonferroni, were not applied as the purpose was exploratory, rather than seeking to make significant claims. Weak correlations ($r<0.6$) were observed, and most of the correlations were found with the IMU features. It should be noted that the number of IMU features was more than 1.5 times of the number of Touch features (i.e. 164 IMU features vs. 105 Touch features). Nevertheless, some variables were only correlated with the Touch features, including in the TD group, MABC-2 manual dexterity and FAB-GTC gestures to command scores; in the ASD group, MABC-2 aiming and catching, and manual dexterity scores; and in the DCD group, the verbal IQ scores.

Neuroimaging Results

Main effects. During the execution and imitation tasks, across all groups and all conditions, there was widespread significant activation, including regions in the bilateral superior frontal cortex, precentral gyrus, postcentral gyrus, inferior frontal gyrus, premotor cortex, superior parietal cortex, cingulate cortex, lateral occipital regions, cerebellum, insular cortex, and STS (Figure 2A).
